# Supplementary material for: Measurement properties of the sit-to-stand test in people with chronic obstructive pulmonary disease: Protocol for a systematic review and meta-analysis using the COSMIN guidelines
Source: PLoS One. 2024 Dec 30;19(12):e0316451. doi: 10.1371/journal.pone.0316451 (PMC11684672; doi:10.1371/journal.pone.0316451)
Supplement: S2 Table — (PDF) [file pone.0316451.s002.pdf]

Measurement properties of the sit-to-stand test in people with chronic obstructive pulmonary disease: Protocol for a systematic review and meta-analysis using the COSMIN guidelines

Supplemental File 2

Christopher Farley[a]; Stuart M. Phillips[b¶]; Jenna Smith-Turchyn[a¶]; Dina Brooks[a,c*]

[a] School of Rehabilitation Science, Faculty of Health Science, McMaster University, Hamilton ON, Canada

[b] Department of Kinesiology, McMaster University, Hamilton ON, Canada

[c] Department of Respiratory Medicine, West Park Healthcare Centre, Toronto ON, Canada

S2 Table. Preliminary MEDLINE search strategy

**1.** (instrumentation or methods).sh.
**2.** (validation study or comparative study).pt.
**3.** exp Psychometrics/
**4.** psychometr*.tw.
**5.** (clinimetr* or clinometr*).mp.
**6.** exp Outcome Assessment, Health Care/
**7.** outcome assessment.tw.
**8.** outcome measure*.mp.
**9.** exp Observer Variation/
**10.** observer variation.tw.
**11.** exp Health Status Indicators/
**12.** exp Reproducibility of Results/
**13.** reproducib*.tw.
**14.** exp Discriminant Analysis/
**15.** (reliab* or unreliab* or valid* or coefficient of variation or coefficient or homogeneity or homogeneous or internal consistency).tw.
**16.** (cronbach* and (alpha or alphas)).tw.
**17.** (item and (correlation* or selection* or reduction*)).tw.
**18.** agreement.mp.
**19.** precision.mp.
**20.** imprecision.mp.
**21.** precise values.mp.
**22.** test-retest.tw.
**23.** (test and retest).tw.
**24.** (reliab* and (test or retest)).tw.
**25.** stability.tw.
**26.** (interrater or inter-rater or intrarater or intra-rater).tw.
**27.** (intertester or inter-tester or intratester or intra-tester).tw.
**28.** (interobserver or inter-observer or intraobserver or intra-observer).tw.
**29.** (intertechnician or inter-technician or intratechnician or intra-technician).tw.
**30.** (interexaminer or inter-examiner or intraexaminer or intra-examiner).tw.
**31.** (interassay or inter-assay or intraassay or intra-assay).tw.
**32.** (interindividual or inter-individual or intraindividual or intra-individual).tw.
**33.** (interparticipant or inter-participant or intraparticipant or intra-participant).tw.
**34.** kappa.tw.
**35.** kappas.tw.

36. repeatab*.mp.

37. ((replicab* or repeated) and (measure or measures or findings or result or results or test or tests)).mp.

38. (generaliza* or generalisa*).tw.

39. concordance.tw.

40. (intraclass and correlation*).tw.

41. discriminative.tw.

42. known group.tw.

43. (factor analysis or factor analyses or factor structure or factor structures).tw.

44. dimension*.tw.

45. subscale*.tw.

46. (multitrait and scaling and (analysis or analyses)).tw.

47. item discriminant.tw.

48. interscale correlation*.tw.

49. (error or errors).tw.

50. individual variability.tw.

51. interval variability.tw.

52. rate variability.tw.

53. (variability and (analysis or values)).tw.

54. (uncertainty and (measurement or measuring)).tw.

55. standard error of measurement.tw.

56. sensitiv*.tw.

57. responsive*.tw.

58. (limit and detection).tw.

59. minimal detectable concentration.tw.

60. interpretab*.tw.

61. ((minimal or minimally or clinical or clinically) and (important or significant or detectable) and (change or difference)).tw.

62. (small* and (real or detectable) and (change or difference)).tw.

63. meaningful change.tw.

64. ceiling effect.tw.

65. floor effect.tw.

66. item response model.tw.

67. IRT.tw.

68. rasch.tw.

69. differential item functioning.tw.

70. DIF.tw.

71. computer adaptive testing.tw.

72. item bank.tw.

73. cross-cultural equivalence.tw.

74. 1 or 2 or 3 or 4 or 5 or 6 or 7 or 8 or 9 or 10 or 11 or 12 or 13 or 14 or 15 or 16 or 17 or 18 or 19 or 20 or 21 or 22 or 23 or 24 or 25 or 26 or 27 or 28 or 29 or 30 or 31 or 32 or 33 or 34 or 35 or 36 or 37 or 38 or 39 or 40 or 41 or 42 or 43 or 44 or 45 or 46 or 47 or 48 or 49 or 50 or 51 or 52 or 53 or 54 or 55 or 56 or 57 or 58 or 59 or 60 or 61 or 62 or 63 or 64 or 65 or 66 or 67 or 68 or 69 or 70 or 71 or 72 or 73

75. exp pulmonary disease, chronic obstructive/ or exp bronchitis, chronic/ or exp pulmonary emphysema/

76. COPD.mp.

77. (obstruct* adj3 (pulmonary or lung* or airway* or airflow* or bronch* or respirat*)).mp.

78. (Chronic adj2 bronchitis).mp.

79. emphysema.mp.

80. 75 or 76 or 77 or 78 or 79

81. ((sit or sitting or rise or rising) adj5 (stand or standing)).mp.

82. ((stand or standing) adj up).tw.

83. ((stand or standing or rise or rising or getting up) adj5 (seat or seated or chair or sit*)).tw.

84. STS.mp.

85. (STST or 30sSTS or 30STS or 5STS or 60STS or 60sSTS or 1mSTS or 1minSTS or 3mSTS or 3minSTS or 2mSTS or 2minSTS or 12STS or 15STS or 20STS).mp.

86. 81 or 82 or 83 or 84 or 85

87. 74 and 80 and 86
